# Cross-Sectional and Longitudinal Associations Between the Serum G\lobulin Level, and Renal Impairment and All-Cause Deaths in Chinese Patients With Newly Diagnosed Multiple Myeloma

**DOI:** 10.3389/fonc.2022.850961

**Published:** 2022-06-08

**Authors:** Jun Cheng, Jia Sun, Yi Zhao, Xiayu Li, Yan Jiang, Rong Lv, Heng Li, Jianghua Chen

**Affiliations:** ^1^ Kidney Disease Center, The First Affiliated Hospital, Medical School of Zhejiang University, Hangzhou, China; ^2^ Department of Kidney Disease, First People’s Hospital of Linping District, Hangzhou, China; ^3^ Hematology, The First Affiliated Hospital, Medical School of Zhejiang University, Hangzhou, China

**Keywords:** Serum Globulin Level, renal impairment, All-cause deaths, Multiple Myeloma, Chinese Patients

## Abstract

**Background:**

A large number of studies have shown that serum globulin plays an important role in a variety of cancers; However, few studies have identified the association between serum globulin levels and end-stage renal disease (ESRD) and all-cause death in Chinese patients with multiple myeloma (MM).

**Methods:**

A generalized additive model and smooth curve fitting were fitted to assess the cross-sectional relationship between the serum globulin levels and renal impairment (RI) at baseline. Multivariate-adjusted Cox regression models were performed to determine the associations between the baseline serum globulin levels and the onset of all-cause death and ESRD in patients with MM.

**Results:**

288 participants who were followed for > 3 months were eligible for the retrospective study. The median serum globulin level was 5.1 ± 2.6 mg/dL. The average follow-up time was 23.3 months. Thirty-two patients (11.5%) had ESRD and 24 patients (8.33%) died after diagnosis. In patients with a serum globulin level < 6.1 mg/dL, the serum globulin level had an independent, negative correlation with the occurrence of MM-related RI. Patients were divided into three groups on the basis of serum globulin tertiles: low (L group), 3.3 mg/dL; middle (M group), 3.3–6.0 mg/dL; and high (H group), 6.0 mg/dL. Cox regression analysis showed that low serum globulin levels may be independent risk factors for all-cause death and the occurrence of ESRD in patients with MM; however, an elevated baseline serum globulin can predict all-cause deaths in patients with MM, but cannot predict the onset of ESRD.

**Conclusions:**

This observational study suggested that there was a non-linear relationship between the serum globulin level and the occurrence of RI in patients with MM. This finding showed that the serum globulin level had a U-shaped association with all-cause death and an L-shaped association with ESRD in patients with MM.

## Introduction

Multiple myeloma (mm) is a clonal B-cell malignant tumor of bone marrow that is related to a variety of clinical manifestations, including renal impairment (RI), hypercalcemia, bone disease and anemia.MM is the second most common hematological malignant tumor, accounting for 1% of all malignancies (1).

RI is a common clinical complication of MM. When defined as serum creatinine levels exceeding 2 mg/dL, 15%-40% of MM patients had RI (2). Indeed, RI can predict the poor prognosis of MM patients. In fact, the latest research from Ireland (3) and the United Kingdom (4) have shown that despite the availability of new drugs, the survival rate of dialysis-dependent patients in the first few weeks after the diagnosis of MM has not significantly improved. Therefore, it is important to identify simple and reliable biomarkers to predict death and identify patients at high risk of renal failure.

The two main components of serum, albumin (ALB) and globulin (GLB), are usually measured by biochemical tests. Albumin reflects the nutritional status of patients and globulin plays a key role in immunity and inflammation (5–8). An abnormal serum globulin level is a common clinical finding in newly diagnosed MM. One study detected hyper- and hypo-gammaglobulinemia in 49.2% and 32.8% of patients, respectively (9). This study showed that more than one-half of MM patients had abnormal serum globulin levels. In fact, some patients are diagnosed with MM because of an increase in the serum globulin level.

Serum globulin is recognized as a prognostic indicator for many cancers, including gastric, colorectal, breast, ovarian, and nasopharyngeal cancers (10–12). Renal damage and an abnormal serum globulin level are common clinical manifestations of MM; however, the association between the serum globulin level, and ESRD and all-cause deaths in patients with MM has not been elucidated.

Therefore, we conducted a retrospective study to determine the association between MM-related RI and the serum globulin level through cross-section analysis. We also determined whether an elevated serum globulin level predicts renal failure and all-cause deaths in a cohort of MM patients followed for exceeding 3 months.

## Subjects and Methods

In this retrospective study, we collected clinical data of newly diagnosed MM patients from the First Affiliated Hospital of Zhejiang University School of Medicine, China, from January 2011 to June 2017.Our study was approved by the Ethics Committee of Hospital (reference number: 20191380). The diagnosis of MM was defined according to the International Myeloma Working Group (IMWG) criteria (13). The exclusion criteria were as follows: 1) a history of other solid tumors, severe infection, kidney disease, severe infection, or liver disease; 2) missing important clinical data, such as serum globulin and albumin levels prior to chemotherapy; and 3) follow-up time < 3 months.

### Data Collection and Laboratory Measurements

Clinical data and baseline demographic characteristics were retrieved electronically from medical records in the general hospital registry. For MM patients admitted to the hospital multiple times, only the first set of observation data is recorded.

The following indicators were evaluated: 1) demographic characteristics, including underlying disease (hypertension and diabetes), gender, and age; 2) laboratory data, including serum calcium, serum globulin, hemoglobin, serum creatinine, serum albumin, serum/urine light chain protein, serum beta-2 microglobulin,and lactate dehydrogenase (LDH); 3) duration of follow-up; 4) anti-myeloma therapy regimen; and 5) time of death, or dialysis and serum creatinine at the last follow-up evaluation.

### Investigation of Study Outcomes

First, we conducted a cross-sectional study to determine the association between the serum globulin level and RI. According to the new IMWG symptomatic MM standard, the definition of RI is based on increased SCR greater than 2 mg/dL or reduced creatinine clearance (eGFR< 40ml/min). Evaluation of the eGFR was assessed by the CKD-EPI equation from Chronic Kidney Disease Epidemiology Collaboration (13–15).

We also determined whether an elevated serum globulin level predicts all-cause deaths and kidney disease outcomes. If the patient were not lost to follow-up or die within 3 months after the designated follow-up time, the information before the final record was used.

The primary outcome was the onset of ESRD (defined as an eGFR less than 15 mL/min or the initiation of renal replacement therapy) or death.

### Statistical Analysis

Categorical variables are presented as a percentage or number. Continuous data are expressed as the median or mean ± standard deviation. The study patients were divided into three groups by the basis of serum globulin tertiles. The difference between three groups was assessed using the chi-squared test, Student’s *t*-test, or the Mann–Whitney *U*-test, as indicated.

Then, we used a multivariable linear regression model to assess the independent association between the presence of RI and serum globulin level at baseline and adjusted for potential confounding factors. A generalized additive model and smooth curve fitting (penalized spline method) were used to estimate the relationship between the serum globulin level, and RI at baseline. We further made use of a two-piecewise linear regression model to investigate the non-linear relationship. If there was a non-linear correlation, a two-piecewise linear regression model was used to estimate the threshold effect of serum globulin levels on RI based on the smoothed graph. When the threshold level was obvious on the smooth curve, the inflection point is automatically estimated by recursive method, and the maximum likelihood model was used (16, 17).

A Cox proportional hazards model was used to study independent variables for the primary end-point. The results are expressed as a hazard ratio (HR) and 95% confidence interval (CI). All probabilities are two-tailed, and statistical analysis used Empower Stats (www.empowerstats.com; X & Y Solution, Inc., Boston, MA, USA) and R software (http://www.R-project.org) (17). A p-value < 0.05 was considered statistically significant.

## Results

Of the 603 participants, 38 were excluded from the study; Therefore, a total of 565 patients were included in this study. The mean age was 63.1 ± 10.1 years. Of the 565 patients with newly diagnosed MM, 61.1% men. According to IMWG criteria, 172 (30.4%) of 565 newly diagnosed MM patients had RI(18).

Two hundred and seventy-seven patient (277) were lost to follow-up because they stopped treatment or returned to local hospitals for further treatment. Two hundred and eighty-eight (288) patients with newly diagnosed MM were followed for > 3 months. RI occurred in 68 (23.6%) of 288 newly diagnosed MM patients.

The median serum globulin level was 5.1 ± 2.6mg/dL. Hypergammaglobulinemia (> 4 mg/dL) was noted in 58.44% of newly diagnosed MM patients, but the incidence of hypogammaglobulinemia (< 2 mg/dL) was 9.38% of newly diagnosed MM patients.

The mean duration of follow-up was 23.3 months. Twenty-four patients (8.33%) died and 32 patients (11.5%) had ESRD after diagnosis. The patients were divided into three groups on the basis of serum globulin tertiles:low (L group), 3.3 mg/dL; middle (M group), 3.3–6.0 mg/dL; and high (H group), 6.0 mg/dL.

**Figure 1:**
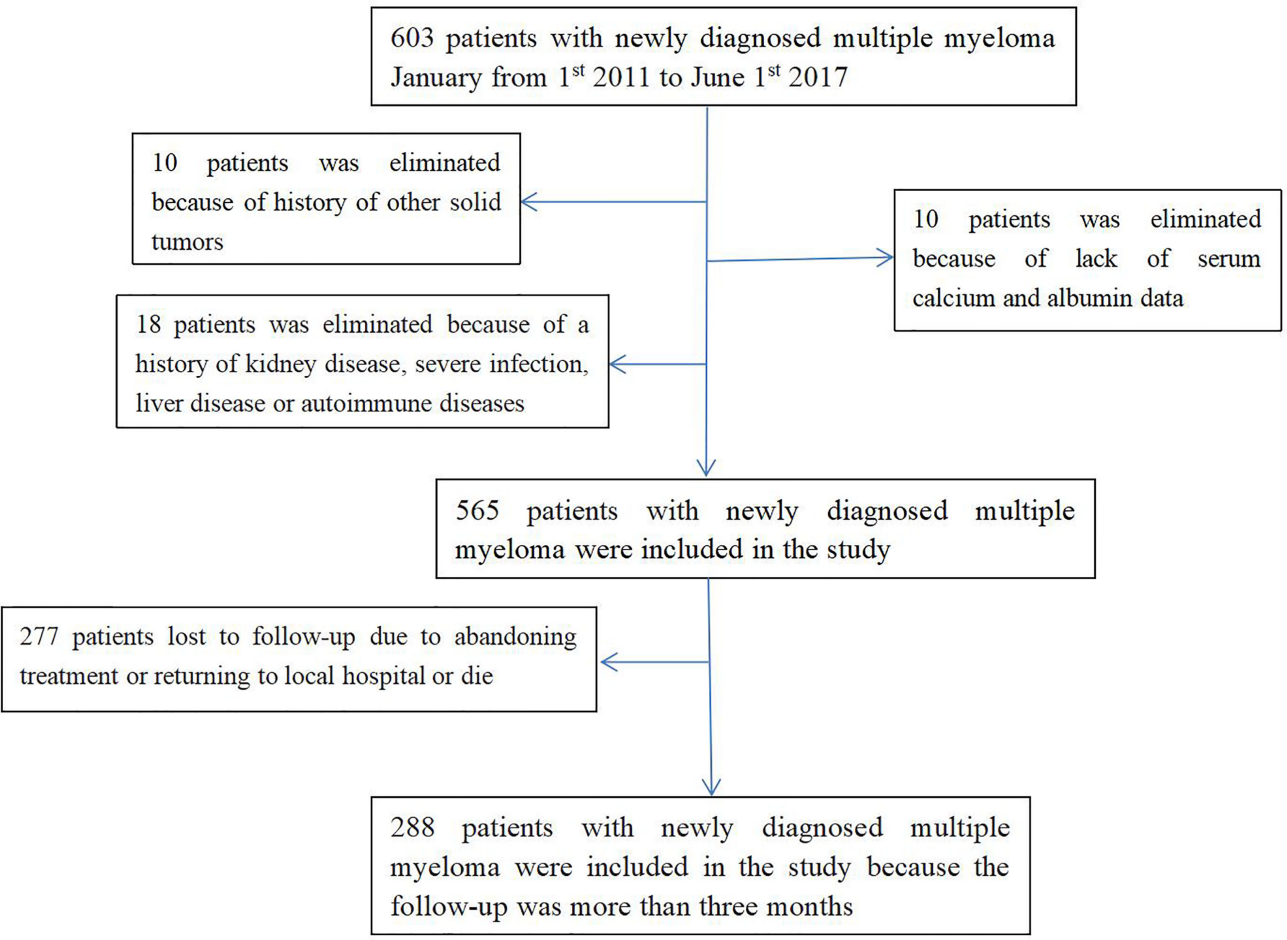
Flow chart of participants in the cohort. 565 patients were enrolled were enrolled in the cross-sectional study. Of these, 277 patients were excluded and 288 patients with newly diagnosed MM were followed for > 3 months were enrolled in the longitudinal study. MM, multiple myeloma.

The baseline characteristics of the study population are listed in **Table 1**. The details of exclusion and enrollment are shown in **Figure 1**.

**Table 1:** Baseline clinical and fifindings of the studied patients separated by tertiles of serum globulin level.

Variable	Tertiles of serum globulin level(mg/dl)			P-value
	<3.3	>=3.3, <6.0	>=6.0	
	Low group	Middle group	High group	
N	95	95	98	N
Age(year)	60.4 ± 9.3	62.5 ± 9.7	61.5 ± 9.5	0.317
Sex				0.666
Female	40 (42.1%)	34 (35.8%)	39 (39.8%)	
Male	55 (57.9%)	61 (64.2%)	59 (60.2%)	
Follow-up time(month)	22.2 ± 17.9	22.2 ± 16.4	25.5 ± 19.9	0.333
Urinary kappa light chain(mg/dl)	436.1 ± 1061.3	79.6 ± 247.5	115.8 ± 275.7	0.001
Urinary lambda light chain(mg/dl)	705.3 ± 1610.5	151.1 ± 648.8	62.2 ± 207.0	0.001
Hemoglobin(g/l)	102.6 ± 27.9	101.6 ± 23.2	83.5 ± 19.8	0.001
Serum albumin(g/l)	4.2 ± 0.6	3.8 ± 0.7	2.9 ± 0.6	0.001
eGFR(ml/min)	56.2 ± 41.8	82.7 ± 37.2	73.8 ± 28.6	0.001
Serum kappa light chain(mg/dl)	744.3 ± 652.2	1947.2 ± 1973.1	3980.8 ± 4281.5	0.001
Serum lambda light chain(mg/dl)	555.3 ± 562.7	1223.4 ± 1479.8	2307.3 ± 2978.3	0.001
serum globulin(mg/dl)	2.3± 0.5	4.6 ± 0.7	8.19 ± 1.48	0.001
Lactate dehydrogenase(u/l)	233.3 ± 131.9	180.7 ± 68.8	165.6 ± 78.7	0.001
Serum beta 2 microglobulin(ug/l)	8751.9 ± 8081.7	5551.9 ± 5421.1	7074.1 ± 6065.6	0.007
ISS-Stage				<0.001
I	28 (33.3%)	31 (34.1%)	7 (7.2%)	
II-III	56 (66.7%)	60 (65.9%)	90 (92.8%)	
Antimyeloma Therapy				0.287
Non-Bortezomib group	31 (33.0%)	40 (42.6%)	32 (33.0%)	
Bortezomib group	63 (67.0%)	54 (57.4%)	65 (67.0%)	
Renal impairment				0.001
No	56 (58.9%)	80 (84.2%)	84 (85.7%)	
Yes	39 (41.1%)	15 (15.8%)	14 (14.3%)	
ESRD				0.001
No	71 (76.3%)	90 (95.7%)	92 (93.9%)	
Yes	22 (23.7%)	4 (4.3%)	6 (6.1%)	
All-cause death				0.007
No	84 (88.4%)	94 (98.9%)	86 (87.8%)	
Yes	11 (11.6%)	1 (1.1%)	12 (12.2%)	

### Relationship Between the Serum Globulin Level and the Occurrence of RI Based on Cross-Section Analysis

Based on multivariable linear regression analysis adjusted for relevant confounding variables, serum globulin levels showed an independent correlation with RI (**Table 2**).

**Table 2:** Cross-sectional Correlation Analyses between serum globulin level and the presence of RI in different models.

Variable	The occurrence of RI		
	Crude model (HR, 95%CI, P)	Minimally adjusted model (HR, 95%CI, P)	Fully adjusted model (HR, 95%CI, P)
serum globulin(mg/dl)	0.8 (0.7, 0.9) <0.001	0.8 (0.7, 0.9) <0.001	0.7 (0.5, 1.0) 0.034
serum globulin level			
L group	1.0	1.0	1.0
M group	0.3 (0.1, 0.6) <0.001	0.2 (0.1, 0.5) <0.001	0.2 (0.0, 0.5) 0.002
H group	0.2 (0.1, 0.5) <0.001	0.2 (0.1, 0.5) <0.001	0.1 (0.0, 0.3) <0.001

Crude model adjust for: None.

Minimally adjusted model:adjust for: age; sex.

Fully adjusted model adjust for: age; sex; hypertension history; diabetes history,LDH, HB, serum albumin, serum/urinary kappa light chain, ISS-stage, serum/urinary lambda light chain and serum beta 2 microglobulin.

**Figure 2:**
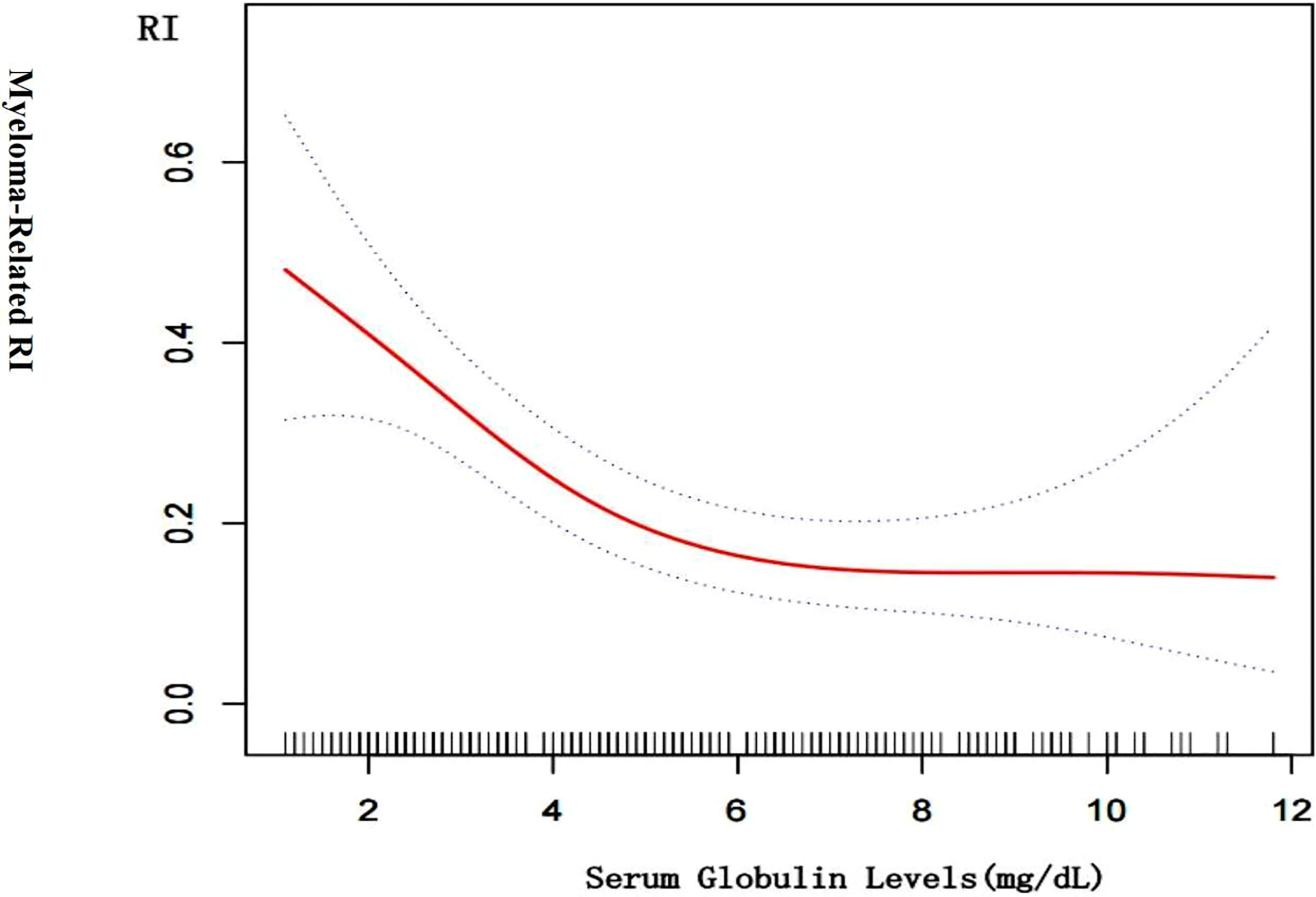
Cross-sectional associations of serum globulin level with by using in a generalized additive model and smooth curve fitting. There was a negative correlation between the serum globulin level and RI (p < 0.01) after adjusting for age; sex, LDH, urinary kappa/ Lambda light chain, serum kappa/Lambda light chain, HB, ISS-stage, serum albumin, serum beta 2 microglobulin.

**Figure 2** is a smoothing plot of the serum globulin level versus RI. The curve showed that the relationship between serum globulin level and RI was not a simple linear relationship. Specifically, as shown in **Table 3**, threshold effect analysis showed that the incidence of RI decreased as the serum globulin level increased up to 6.1 mg/dL [0.6 (0.4, 0.8), <0.001]. In patients with a serum globulin level > 6.1 mg/dL, the serum globulin level was not significantly associated with RI in MM patients [0.9 (0.7, 1.3), 0.709]. In patients with a serum globulin level < 6.1 mg/dL, the correlation coefficient of RI was positive [β = 6.2, p < 0.001; 0.6 (0.4, 0.8)].

**Table 3:** Threshold effect analysis of serum globulin level and the presence of RI using piece-wise linear regression.

Model	Result [β (95%CI) P value]
Model I	
one-line linear regression model	0.7 (0.5, 0.9) 0.003
Model II	
turning point	6.1 mg/dl
Group1<6.1 correlation coefficient (β1)	0.6 (0.4, 0.8) <0.001
Group2 >6.1, correlation coefficient (β1)	0.9 (0.7, 1.3) 0.709
predictive value of RI at turning point	
a log likelihood ratio test	1.6 (1.1, 2.5) 0.026

Effect: serum globulin level; Cause: myeloma-related RI.

adjusted for age; sex; hypertension history; diabetes history LDH, ISS-stage, serum albumin, serum/urinary lambda light chain, serum/urinary kappa light chain, HB, serum beta 2 microglobulin.

### Associations Between Baseline Serum Globulin Level and All-Cause Deaths

In a multivariable proportional hazards Cox regression analysis adjusted according to demographic and clinical factors, we selected age, gender, eGFR,HB,LDH,RI, albumin, serum/urinary lambda or kappa light chains, serum beta 2-microglobulin, and anti-myeloma therapy regimen as confounding factors.

We calculated the HRs for the progression of all-cause deaths with 95% CIs using the age-and gender-adjusted model (model 1), and a model fully adjusted for demographic and clinical confounders (model 2) for the L and H groups vs. the M group (**Table 4**). In model 1 and the model 2, the patients in the H group had a significantly higher risk for all-cause deaths compared with the patients in the M group. The patients in the L group also had a 9.38–fold [95% CI (1.00, 87.70)] higher risk for all-cause deaths than the patients in the M group.

**Table 4:** Relationship between serum globulin level and All-cause death and ESRD in different models.

Variable	All-cause death			ESRD		
	Crude model (HR, 95%CI, P)	Minimally adjusted model(HR, 95%CI, P)	Fully adjusted model(HR,95%CI,P)	Crude model (HR, 95%CI, P)	Minimally adjusted model(HR, 95%CI, P)	Fully adjusted model (HR, 95%CI, P)
serum globulin level						
M group	1.0	1.0	1.0	1.0	1.0	1.0
Low group	11.40 (1.47, 88.39) 0.0199	12.40 (1.58,97.06) 0.0165	9.38 (1.00, 87.70) 0.0496	5.76 (1.98, 16.77) 0.0013	6.53 (2.22, 19.19) 0.0007	5.38 (1.41, 20.56) 0.0140
High group	8.77 (1.13, 68.18) 0.0379	9.38 (1.20, 73.24) 0.0327	14.05 (1.36, 144.53) 0.0263	1.24 (0.35, 4.41) 0.7375	1.38 (0.39, 4.93) 0.6171	2.75 (0.45, 16.63) 0.2718

Crude model adjust for: None.

Minimally adjusted model: adjust for: age sex.; hypertension history; diabetes history.

Fully adjusted model adjust for: age; sex.; hypertension history; diabetes history, serum/urinary lambda light chain, LDH, serum/urinary kappa light chain, HB, ISS-stage, serum albumin and serum beta 2 microglobulin.

**Figure 3:**
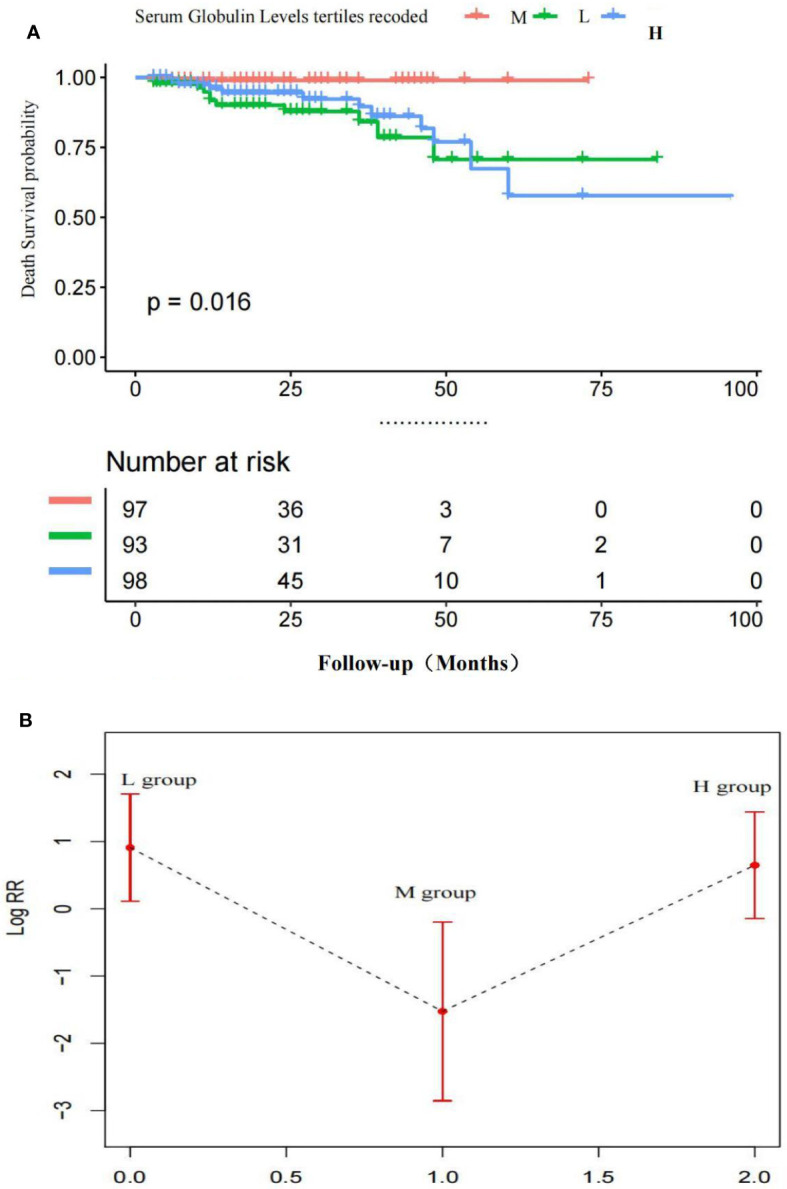
**(A)** Adjusted Kaplan-Meier curves of all-cause death outcome according to serum globulin levels *Survival in the M group was significantly higher than the H group and L group. **(B)** Adjusted Log risk ratios (RRs) for all-cause death outcome by tertiles of serum globulin levels. A U-shaped association was shown to exist between the serum globulin level and all-cause deaths in patients with MM. P < 0.05 M vs. H group; P < 0.05 M vs. L group; P > 0.01 H vs. L group.

**Figure 3A** indicates the adjusted Kaplan–Meier estimates for all-cause deaths and survival in relation to the serum globulin level in each group. Survival was lowest in the H group and survival in the M group was significantly higher than the H group and L group.

**Figure 3B** is a smoothing plot of the serum globulin level versus all-cause deaths. The curve shows that the relationship between the serum globulin level and all-cause deaths was not simply linear. A U-shaped association was shown to exist between the serum globulin level and all-cause deaths in patients with MM.

### Associations Between the Baseline Serum Globulin Level and ESRD

Furthermore, the fully adjusted model, including age, gender, eGFR, HB, LDH, RI, albumin, serum/urinary lambda or kappa light chains, serum beta 2-microglobulin, and anti-myeloma therapy regimen showed that the patients in the L group had a significantly higher risk for the development of ESRD compared with the M group. The patients in the H group had a 2.75-fold (95% CI: 0.45, 16.63) higher risk for ESRD than the M group in model 2, but this effect did not reach statistical significance (P = 0.08). The patients in the L group had a 5.38–fold [95% CI (1.41, 20.56)] higher risk for ESRD than the patients in the M group.

**Figure 4:**
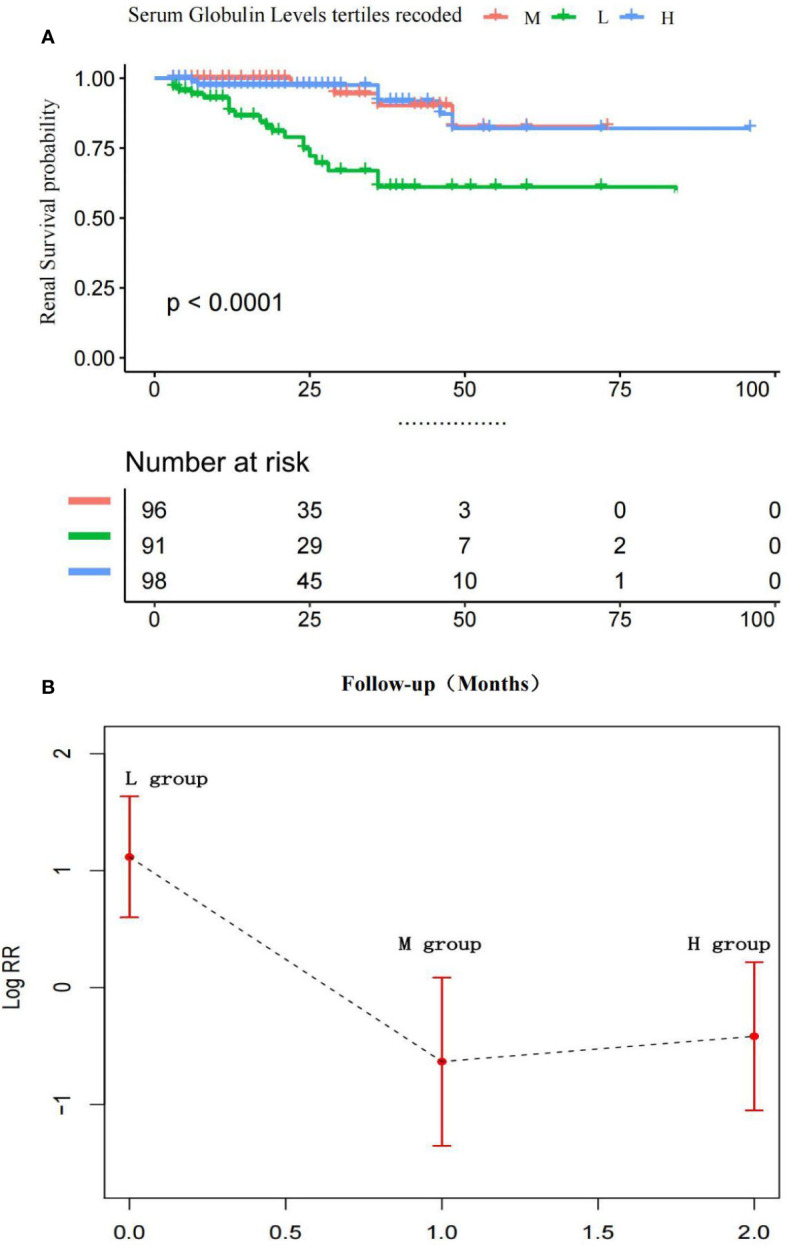
**(A)** Adjusted Kaplan-Meier curves of ESRD outcome according to serum globulin levels.Renal survival was lowest In the L group, Renal survival analysis between the H and M groups was not significantly different. P > 0.01 M vs. H group; P < 0.01 M vs. L group and H vs. L group. **(B)** Adjusted Log risk ratios (RRs) for ESRD by tertiles of serum globulin levels. A L-shaped association was shown to exist between the serum globulin level and ESRD in patients with MM.

**Figure 4A** indicates the adjusted Kaplan–Meier estimates for renal survival in relation to the serum globulin level in each group. In MM patients, renal survival was lowest in the L group. Renal survival analysis between the H and M groups was not significantly different, as determined by adjusted analysis (log-rank test, P = 0.74).

**Figure 4B** is a smoothing plot of the serum globulin level versus ESRD. The curve shows that the relationship between the serum calcium level and ESRD was not simply linear. An L-shaped association between the serum globulin level, and renal outcome and ESRD in patients with MM was demonstrated.

## Discussion

As a malignant tumor with a high incidence, MM always has the characteristics of delayed diagnosis and poor prognosis. RI is a common feature in patients with MM and may provide a clue to the diagnosis and serve as a major medical management issue. This complication occurs in 20%–40% of newly diagnosed patients with MM. The 2-year all-cause mortality of patients with ESRD due to MM-related RI is 58% versus 31% in all other patients without RI (19).

Early mortality is defined as death by any cause within the first 6 months after the pathologic diagnosis of MM (20). Early mortality after diagnosis of MM is usually attributed to the combined effects of active diseases and comorbidity factors. Recent population studies have shown that nearly a quarter of MM patients die within 1 year after diagnosis, and nearly half of the deaths occur within the first 3 months (21, 22).

Infection and renal failure are the main direct causes of early death, which cannot be accurately predicted by prognostic characteristics (20–22). Therefore, patients with relatively stable conditions were selected in this study, and the patients included in the study completed at least three cycles of chemotherapy.

The prognosis of MM may be affected by many factors, such as age, gender, hypercalcemia, LDH, free light chain and RI (23). These factors may serve as predictors for the prognosis of patients with MM; however, these factors alone are not sufficient to make an accurate prediction because many other factors related to the host and tumor must be considered. At the same time, we should also know that due to unbalanced economic development, many hospitals do not routinely detect free light chains and M protein quantification, and clinicians need simpler and more direct parameters to judge the prognosis of patients.

In this study, we determined the association of the serum globulin level, with ESRD and all-cause deaths in Chinese patients with newly diagnosed MM using a retrospective cohort. In this study we demonstrated that not only an elevated serum globulin level, but also a low serum globulin level are significant risk factors for the development of all-cause deaths. A U-shaped association between the serum globulin level and an L-shaped association between renal outcome in patients with MM were shown to exist.

Globulin, including gamma antibodies or glycoprotein and globulins, is one the most important factors of blood proteins (24). Globulin can act as a regulator in the circulatory system by helping blood clot, transporting proteins through lipoproteins, and indicating antibody levels. Elevated globulin levels can be attributed to chronic inflammatory diseases, liver disease, autoimmune conditions, ulcerative colitis, and kidney disease (25–27). Immunoglobulins are an important component of serum globulin. The immunoglobulins include five types of antibodies (IgG, IgA, IgM, IgD, and IgE) that consist of heavy and light chains connected by disulfide bonds. Monoclonal immunoglobulins can consist of light chains (κ or λ) alone, or rarely heavy chains alone(28).

In addition to inflammatory states, elevated MM globulin is derived from clones of abnormal plasma cells. The growth of plasma cells in the bone marrow leads to the production of monoclonal immunoglobulins. Increased proliferation of monoclonal immunoglobulins can lead to increased serum globulin. An elevated serum globulin level with a high proportion of monoclonal immunoglobulins often corresponds to a high tumor burden (28). In this study we demonstrated that an elevated serum globulin level is a significant risk factor for the development of all-cause deaths in patients.

Myeloma associated renal disease is partially caused by free light chain deposition in the renal tubules (14). Immunoglobulins and other acute phase proteins, such as serum amyloid A, CRP, complement C3, fibrinogen, and ceruloplasmin, are part of the calculated globulin fraction and play a key role in the immune and inflammatory cascade (25–27). Gao et al. (29) reported that hypogammaglobulinemia in patients with symptomatic MM at the time of diagnosis is an independent poor prognostic factor. In fact, a decreased serum globulin level often indicates more severe hypogammaglobulinemia and immunoparesis for MM patients. In the current study, we also showed that a low serum globulin level is a significant risk factor for all-cause deaths and ESRD in patients.

Our study had several limitations. First, observational design, retrospective recording of serum creatinine levels, serum globulin levels at a single time point in the late course of newly diagnosed MM, and potential misclassification of study measurements may affect the results. Second, after a clear diagnosis of MM was established, some patients were followed for < 3 months, so they were not included in this study because some patients returned to the local hospital for treatment. The sample size of this study is relatively small, which may lead to some small sample deviations and limit the statistical power. Therefore, it is necessary to conduct large-scale studies involving more patients with prospective design in the future.

## Conclusions

In conclusion, our study showed that the baseline serum globulin level of newly diagnosed MM patients in China was independently correlated with RI. There was a non-linear relationship between the serum globulin level and RI. A low serum globulin level may be an independent risk factor for all-cause deaths and the occurrence of ESRD in patients with MM; However, an elevated baseline serum globulin predicted all-cause deaths in patients with MM, but did not predict the occurrence of ESRD. The study suggested that not only an elevated serum globulin but also a low serum globulin level may serve as a useful clinical biomarker for the survival of MM patients followed for > 3 months. Our findings were hypothesis-generating based on the results of a retrospective observational study. Therefore, further studies with more patients are needed to validate our findings.

## Data Availability Statement

The raw data supporting the conclusions of this article will be made available by the authors, without undue reservation.

## Ethics Statement

In this retrospective study, we collected clinical data of newly diagnosed MM patients from the First Affiliated Hospital of Zhejiang University School of Medicine, China, from January 2011 to June 2017.Our study was approved by the Ethics Committee of Hospital (reference number: 20191380).

## Author Contributions

This study was designed and supervised by JHC and JC. Data analyses were performed by YZ, JS, XL, RL, HL and YJ. All authors contributed to the article and approved the submitted version.

## Funding

This work was supported by grant LY19H050007 and LQ19H050009 from Zhejiang Provincial Natural Science Foundation of China and grant 2016KYA087 from the Zhejiang Medical and Health Science and Technology Project.

## Conflict of Interest

The authors declare that the research was conducted in the absence of any commercial or financial relationships that could be construed as a potential conflict of interest.

## Publisher’s Note

All claims expressed in this article are solely those of the authors and do not necessarily represent those of their affiliated organizations, or those of the publisher, the editors and the reviewers. Any product that may be evaluated in this article, or claim that may be made by its manufacturer, is not guaranteed or endorsed by the publisher.

## References

[1] Ludwig H Miguel JS Dimopoulos MA Palumbo A Garcia Sanz R Powles R. International Myeloma Working Group Recommendations for Global Myeloma Care. Leukemia (2014) 28:981–92. doi: 10.1038/leu.2013.293 24177258

[2] Knudsen LM Hjorth M Hippe E. Renal Failure in Multiple Myeloma: Reversibility and Impact on the Prognosis. Nordic Myeloma Study Group. Eur J Haematol (2000) 65:175–81. doi: 10.1034/j.1600-0609.2000.90221.x 11007053

[3] Murphy PT Baldeo C O'Kelly P Sargant J Thornton P McCloy M. Dialysis-Dependent Renal Failure at Diagnosis Continues to be Associated With Very Poor Outcome in Multiple Myeloma. Br J Haematol (2014) 165(6):890–1. doi: 10.1111/bjh.12818 24593674

[4] Haynes RJ Read S Collins GP Darby SC Winearls CG. Presentation and Survival of Patients With Severe Acute Kidney Injury and Multiple Myeloma: A 20-Year Experience From a Single Centre. Nephrol Dial Transpl (2010) 25(2):419–26. doi: 10.1093/ndt/gfp488 19767634

[5] McPherson R Pincus MR. Henry’s Clinical Diagnosis and Management by Laboratory Methods. 22nd edn. Saunders, Philadelphia: ELSEVIER (2011).

[6] Gabay C Kushner I. Acute-Phase Proteins and Other Systemic Responses to Inflammation. N Engl J Med (1999) 340(6):448–54. doi: 10.1056/NEJM199902113400607 9971870

[7] Huang R Ma Y Holm R Trope CG Nesland JM Suo Z. Sex Hormone-Binding Globulin (SHBG) Expression in Ovarian Carcinomas and its Clinicopathological Associations. PloS One (2013) 8(12):e83238. doi: 10.1371/journal.pone.0083238 24386165 PMC3873286

[8] Kristal AR Schenk JM Song Y Arnold KB Neuhouser ML Goodman PJ. Serum Steroid and Sex Hormonebinding Globulin Concentrations and the Risk of Incident Benign Prostatic Hyperplasia: Results From the Prostate Cancer Prevention Trial. Am J Epidemiol (2008) 168(12):1416–24. doi: 10.1093/aje/kwn272 PMC2727187 18945688

[9] Hussain A Almenfi HF Almehdewi AM Hamza MS Bhat MS Vijayashankar NP. Laboratory Features of Newly Diagnosed Multiple Myeloma Patients. Cureus (2019) 11(5):e4716. doi: 10.7759/cureus.4716 31355076 PMC6650180

[10] Zhao HR Jiang T Tian YY Gao Q Li Z Pan Y. Angiotensin II Triggers Apoptosis *via* Enhancement of NADPH Oxidase-Dependent Oxidative Stress in a Dopaminergic Neuronal Cell Line. Neurochemical Res (2015) 40:854–63. doi: 10.1007/s11064-015-1536-y 25666589

[11] Zhang W Zhangyuan G Wang F Zhang H Yu D Wang J. High Preoperative Serum Globulin in Hepatocellular Carcinoma is a Risk Factor for Poor Survival. J Cancer (2019) 10(15):3494–500. doi: 10.7150/jca.29499 PMC6603401 31293654

[12] Du XJ Tang LL Mao YP Sun Y Zeng MS Kang TB. The Pretreatment Albumin to Globulin Ratio has Predictive Value for Long-Term Mortality in Nasopharyngeal Carcinoma. PloS One (2014) 9:e94473. doi: 10.1371/journal.pone.0094473 24718309 PMC3981820

[13] Masson I Flamant M Maillard N Rule AD Vrtovsnik F Peraldi MN. MDRD Versus CKD-EPI Equation to Estimate Glomerular Filtration Rate in Kidney Transplant Recipients. Transplantation (2013) 95:1211–7. doi: 10.1097/TP.0b013e318288caa6 23511243

[14] Rajkumar SV Dimopoulos MA Palumbo A Blade J Merlini G Mateos MV. International Myeloma Working Group Updated Criteria for the Diagnosis of Multiple Myeloma. Lancet Oncol (2014) 15(12):e538–48. doi: 10.1016/S1470-2045(14)70442-5 25439696

[15] Inker LA Schmid CH Tighiouart H Eckfeldt JH Feldman HI Greene T. Estimating Glomerular Filtration Rate From Serum Creatinine and Cystatin C. N Engl J Med (2012) 367:20–9. doi: 10.1056/NEJMoa1114248 PMC4398023 22762315

[16] Lyu Y Shah PS Ye XY Warre R Piedboeuf B. Association Between Admission Temperature and Mortality and Major Morbidity in Preterm Infants Born at Fewer Than 33 Weeks’ Gestation. JAMA Pediatr (2015) 169(4):e150277. doi: 10.1080/14767058.20201810229 25844990

[17] Liu S Wang X Lu Y Li T Gong Z Sheng T. The Effects of Intraoperative Cryoprecipitate Transfusion on Acute Renal Failure Following Orthotropic Liver Transplantation. Hepatol Int (2013) 7:901–9. doi: 10.1007/s12072-013-9457-9 PMC7102214 26201928

[18] Cheng J Zhang W Zhao Y Li X Lv R Li H. Association of Serum Calcium Levels With Renal Impairment and All-Cause Death in Chinese Patients With Newly Diagnosed Multiple Myeloma: A Cross-Sectional, Longitudinal Study. Nutr Metab (Lond) (2021) 18(1):19. doi: 10.1186/s12986-020-00525-0 33573678 PMC7879693

[19] Dimopoulos MA Delimpasi S Katodritou E Vassou A Kyrtsonis MC Repousis P Kartasis Z. Significant Improvement in the Survival of Patients With Multiple Myeloma Presenting With Severe Renal Impairment After the Introduction of Novel Agents. Ann Oncol (2014) 25:195–200. doi: 10.1093/annonc/mdt483 24356630

[20] Augustson BM Begum G Dunn JA Barth NJ Davies F Morgan G. Early Mortality After Diagnosis of Multiple Myeloma: Analysis of Patients Entered Onto the United Kingdom Medical Research Council Trials Between 1980 and 2002—Medical Research Council Adult Leukaemia Working Party. J Clin Oncol (2005) 23:9219–26. doi: 10.1200/JCO.2005.03.2086 16275935

[21] Costa LJ Gonsalves WI Kumar SK. Early Mortality in Multiple Myeloma. Leukemia (2015) 29:1616–8. doi: 10.1038/leu.2015.33 25673239

[22] Kristinsson SY Anderson WF Landgren O. Improved Long-Term Survival in Multiple Myeloma Up to the Age of 80 Years. Leukemia (2014) 28:1346–8. doi: 10.1038/leu.2014.23 24418994

[23] Reule S Sexton DJ Solid CA Chen SC Foley RN. ESRD Due to Multiple Myeloma in the United States, 2001-2010. J Am Soc Nephrol (2016) 27(5):1487–94. doi: 10.1681/ASN.2014090876 PMC4849810 26516209

[24] Gelfand EW. Intravenous Immune Globulin in Autoimmune and Inflammatory Diseases. N Engl J Med (2012) 367:2015–25. doi: 10.1056/NEJMra1009433 23171098

[25] Chan WL Carrell RW Zhou A Read RJ. How Changes in Affinity of Corticosteroid-Binding Globulin Modulate Free Cortisol Concentration. J Clin Endocrinol Metab (2013) 98:3315–22. doi: 10.1210/jc.2012-4280 PMC3813945 23783094

[26] Simo R Barbosa-Desongles A Lecube A Hernandez C Selva DM. Potential Role of Tumor Necrosis Factor-Alpha in Downregulating Sex Hormone-Binding Globulin. Diabetes (2012) 61:372–82. doi: 10.2337/db11-0727 PMC3266423 22210320

[27] Stegeman BH Helmerhorst FM Vos HL Rosendaal FR Van Hylckama Vlieg A. Sex Hormone-Binding Globulin Levels are Not Causally Related to Venous Thrombosis Risk in Women Not Using Hormonal Contraceptives. J Thromb Haemostasis JTH (2012) 10:2061–7. doi: 10.1111/j.1538-7836.2012.04878.x 22882730

[28] Morrison T Booth RA Hauff K Berardi P Visram A. Laboratory Assessment of Multiple Myeloma. Adv Clin Chem (2019) 89:1–58. doi: 10.1016/bs.acc.2018.12.001 30797467

[29] Gao W Li J Jian Y Yang G Wu Y Li Y. Immunoparesis in Symptomatic Multiple Myeloma at Diagnosis Affects PFS With Bortezomib-Containing Induction Therapy, But Not ASCT Consolidation. Int J Hematol (2019) 109(2):169–74. doi: 10.1007/s12185-018-2547-7 30311142

